# Use of Machine Learning to Assess the Management of Uncomplicated Urinary Tract Infection

**DOI:** 10.1001/jamanetworkopen.2024.56950

**Published:** 2025-01-31

**Authors:** Noah Jones, Ming-Chieh Shih, Elizabeth Healey, Chen Wen Zhai, Sonali Advani, Aaron Smith-McLallen, David Sontag, Sanjat Kanjilal

**Affiliations:** 1 MIT Media Lab, Massachusetts Institute of Technology, Cambridge; 2 College of Life Sciences and Medicine, National Tsing Hua University, Hsinchu, Taiwan; 3 Program in Health Sciences and Technology, Massachusetts Institute of Technology, Cambridge; 4 Operations Research Center, Massachusetts Institute of Technology, Cambridge; 5 Division of Infectious Diseases, Department of Medicine, Duke University School of Medicine, Durham, North Carolina; 6 Independence Blue Cross, Philadelphia, Pennsylvania; 7 Institute for Medical Engineering and Sciences, Massachusetts Institute of Technology, Cambridge; 8 Department of Population Medicine, Harvard Medical School and Harvard Pilgrim Healthcare Institute, Boston, Massachusetts

## Abstract

**Importance:**

Uncomplicated urinary tract infection (UTI) is a common indication for outpatient antimicrobial therapy. National guidelines for the management of uncomplicated UTI were published in 2011, but the extent to which they align with current practices, patient diversity, and pathogen biology, all of which have evolved greatly in the time since their publication, is not fully known.

**Objective:**

To reevaluate the effectiveness and adverse event profile for first-line antibiotics, fluoroquinolones, and oral β-lactams for treating uncomplicated UTI in contemporary clinical practice.

**Design, Setting, and Participants:**

This retrospective, population-based cohort study used a claims dataset from Independence Blue Cross, which contains inpatient, outpatient, laboratory, and pharmacy claims that occurred between 2012 and 2021, formatted into the Observational Medical Outcomes Partnership (OMOP) common data model. Participants were nonpregnant female individuals aged 18 years or older with a diagnosis of uncomplicated, nonrecurrent UTI at an outpatient setting. Patients must also have been treated with first-line (nitrofurantoin or trimethoprim-sulfamethoxazole), fluoroquinolone (ciprofloxacin, levofloxacin, or ofloxacin), or oral β-lactam (amoxicillin-clavulanate, cefadroxil, or cefpodoxime) antibiotics. Data analysis was performed from November 2021 to August 2024.

**Exposures:**

Patients exposed to first-line antibiotics were assigned to the treatment group, and those exposed to fluoroquinolone or β-lactam treatments were assigned to control groups.

**Main Outcomes and Measures:**

The primary outcome was a composite end point for treatment failure, defined as outpatient or inpatient revisit within 30 days for UTI, pyelonephritis, or sepsis. Secondary outcomes were the risk of 4 common antibiotic-associated adverse events: gastrointestinal symptoms, rash, kidney injury, and *Clostridium difficile* infection.

**Results:**

There were 57 585 episodes of UTI among 49 037 female patients (mean [SD] age, 51.7 [20.1]) years), with prescriptions for first-line antibiotics in 35 018 episodes (61%), fluoroquinolones in 21 140 episodes (37%), and β-lactams in 1427 episodes (2%). After adjustment, receipt of first-line therapies was associated with an absolute risk difference of −1.78% (95% CI, −2.37% to −1.06%) for having a revisit for UTI within 30 days of diagnosis vs fluoroquinolones. First-line therapies were associated with an absolute risk difference of −6.40% (95% CI, −10.14% to −3.24%) for 30-day revisit compared with β-lactam antibiotics. Differences in adverse events were similar between all comparators. Results were identical for models built with an automated OMOP feature extraction package.

**Conclusions and Relevance:**

In this cohort study of patients with uncomplicated UTI derived from a large regional claims dataset, national treatment guidelines published almost 14 years ago continue to recommend optimal treatments. These results also provide proof-of-principle that automated feature extraction methods for OMOP formatted data can emulate manually curated models, thereby promoting reproducibility and generalizability.

## Key Points

**Question:**

Are treatments for uncomplicated urinary tract infection (UTI) recommended in national guidelines published in 2011 still optimal?

**Findings:**

Using a large regional claims dataset for 57 585 episodes of UTI occurring in 49 037 female patients, this cohort study found that guideline-concordant first-line treatments retained their efficacy vs fluoroquinolones and outperformed β-lactam antibiotics with respect to efficacy and adverse events.

**Meaning:**

Even though the national treatment guidelines for uncomplicated UTI were published nearly 14 years ago, these findings suggest that outpatient antimicrobial stewardship programs should continue to encourage clinicians to follow them.

## Teaser

This cohort study examines the effectiveness and adverse event profile for first-line antibiotics, fluoroquinolones, and oral β-lactams for treating uncomplicated urinary tract infection (UTI) in contemporary clinical practice.

## Introduction

Up to 50% of women will experience a urinary tract infection (UTI) in their lifetime,^1^ making it the third most common indication for antibiotic treatment in the US after respiratory tract infection and skin and soft-tissue infections.^2^ Treatment guidelines published by the Infectious Diseases Society of America (IDSA) in 2011 encouraged the use of nitrofurantoin, trimethoprim-sulfamethoxazole, and fosfomycin as first-line treatments for uncomplicated UTI on the basis of their effectiveness and relatively limited adverse event profile.^3,4^ Fluoroquinolones are listed as an alternative option because of their predilection for selecting for multidrug-resistant organisms^5^ and their association with serious adverse events, including *Clostridium difficile* colitis.^6^ Despite this, ciprofloxacin and levofloxacin are still the most commonly used antibiotics in the treatment of UTI, which may reflect the real or perceived threat of antibiotic resistance to the first-line agents.^2^ The guidelines list β-lactams as an inferior alternative because they are associated with reduced treatment effectiveness.^7^

The evidence supporting the IDSA treatment guidelines is based on a small number of randomized clinical trials and observational studies,^7,8,9,10,11^ many of which were completed several decades ago. Although those studies provided important information for policymaking, they were limited in terms of the diversity of patients they recruited and were performed at a time when standards of care and health care–seeking behavior differed greatly from current practice. Furthermore, the pathogen strains in circulation at the time of these studies have likely been replaced by new strains that may have a differential response to drug therapies, regardless of their susceptibility phenotype. Therefore, a re-evaluation of management strategies for uncomplicated UTI could provide useful information for treating clinicians and policymakers. In this study, we sought to estimate treatment effectiveness and adverse events for guideline-concordant and guideline-discordant treatments for UTI using causal inference supported by machine learning applied to a large contemporary claims dataset.

## Methods

### Study Design and Data

We conducted a retrospective cohort study using the claims database from Independence Blue Cross, which contains health-related information for 3 million people living primarily in a 5-county area surrounding Philadelphia, Pennsylvania. The dataset contains inpatient, outpatient, laboratory, and pharmacy claims made between 2012 and 2021. The database is formatted in the Observational Medical Outcomes Partnership (OMOP) common data model (version 5), developed by the Observational Health Data Sciences and Informatics initiative.^12^ Reporting of this study follows the Strengthening the Reporting of Observational Studies in Epidemiology (STROBE) reporting guideline.^13^ This study was deemed exempt by the institutional review board of the Massachusetts Institute of Technology. Informed consent was not needed because the data were deidentified, in accordance with 45 CFR §46.

### Study Population

The analysis cohort consisted of nonpregnant female individuals aged 18 and older with a diagnosis of uncomplicated, nonrecurrent UTI at an outpatient setting. The list of diagnosis codes associated with UTI is provided in eTable 1 in Supplement 1. Patients included in the analysis must also have been treated with 1 of the following 3 classes of antimicrobials within a 7-day period after the diagnosis: (1) nitrofurantoin and trimethoprim-sulfamethoxazole (first-line treatments); (2) ofloxacin, ciprofloxacin, and levofloxacin (fluoroquinolones); or (3) amoxicillin-clavulanate, cefadroxil, and cefpodoxime (β-lactams). Fosfomycin was excluded owing to the low number of treatment events.

We excluded individuals with UTI who received treatment outside the 3 aforementioned antibiotic classes (eg, fluconazole) and individuals treated with more than 1 antibiotic within a 7-day period. In addition, to avoid contamination of previous antibiotic exposures, we excluded patients with antibiotic exposure within 7 days before the date of UTI diagnosis. We also excluded those with recurrent UTI, defined as 2 or more episodes in a 180-day period and 3 or more episodes in a 365-day period, and people with complicated UTI, defined as any male individuals with a UTI diagnosis or female individuals with a predefined list of procedures and diagnoses associated with complicated UTI within 180 days of the diagnosis, or any histories of complicating long-term comorbidities, such as neurogenic bladder, spina bifida, or cancers of the genitourinary tract before the UTI diagnosis. A full list of comorbidities flagged for exclusion can be found in eTable 2 in Supplement 1.

### Outcomes and Censoring

We defined 2 primary end points for the analysis. The first was a composite end point for treatment failure, defined as outpatient or inpatient revisit within 30 days for UTI, pyelonephritis, or sepsis. We performed a subgroup analysis for each subtype of infection and specifically for patients who were admitted within a 30-day period after their initial diagnosis of UTI. The second set of end points involved adverse events, defined as the presence of diarrhea within 15 days of the UTI event, acute kidney injury, or a dermatologic adverse event within 30 days of a UTI event or a diagnosis of *C difficile* infection within 90 days of the UTI event. The conditions and the corresponding codes included in each adverse event category are listed in eTable 3 in Supplement 1. Individuals were right-censored from the analysis if they left the health plan before the observational period of the outcome of interest.

### Confounder Generation

We derived 2 sets of baseline covariates, which served as potential confounders. The first used domain expert knowledge from 2 practicing infectious disease physicians (S.A. and S.K.), and the second was derived from the OMOP-learn coding package. OMOP-learn is a data-driven feature extractor developed in prior work and is specifically designed for use with claims datasets formatted in the OMOP common data model.^14^ The package serves to automatically generate time-windowed covariates.

**Figure 1.**
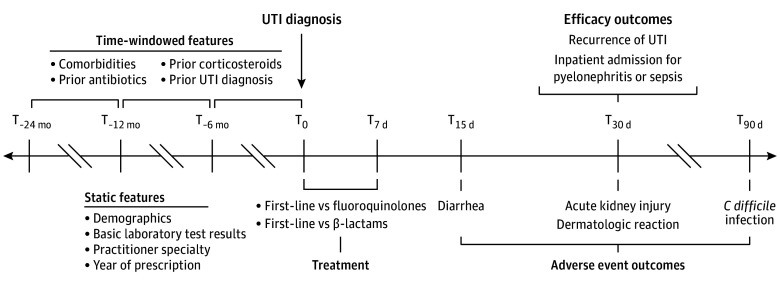
Cohort Inclusion Criteria and Definitions for Outcomes and Features Static features were evaluated at time (T) zero. *C difficile* indicates *Clostridium difficile*; UTI, urinary tract infection.

Domain expert–derived features were classified into demographics, medical conditions, drug prescriptions, UTI history, prior antibiotic exposures, presence of laboratory measurements, physician specialty, and year of prescription to account for secular trends in prescribing behavior. A list of medical conditions and drug prescriptions included in the features is shown in eTable 4 in Supplement 1. Prior antibiotic exposures for treatment groups are shown in eTable 5 in Supplement 1. Domain expert–derived features related to medical histories were binned into nonoverlapping time windows (0 to ≤6 months, >6 to ≤12 months, and >12 to ≤24 months) relative to the date of UTI diagnosis. Laboratory values (urinalysis and blood tests) were counted if they were obtained at the time of the UTI diagnosis. The final expert-derived model consisted of 245 features. OMOP-learn features were derived from diagnoses, procedures, medications, and physician specialties. The total OMOP-derived model consisted of 143 830 features for the comparison between first-line antibiotics and fluoroquinolones and 131 035 features for the comparison between first-line antibiotics and β-lactams. Figure 1 depicts the cohort, outcome, and confounder definitions.

### Statistical Analysis

Data analysis was performed from November 2021 to August 2024. We estimated the absolute risk difference of first-line therapies vs fluoroquinolones or β-lactams for patients with uncomplicated UTI on 30-day recurrence and adverse events. To account for possible covariate-dependent censoring, we used inverse probability of censoring weighting to reweight individuals who were observed or not censored.^15^ In addition, the central problem in estimating antibiotic treatment outcomes is confounding by indication; therefore, we used inverse probability weighted propensity scores to adjust for the likelihood of receipt of each treatment class given an individual’s confounders.

For both the observation probability model and treatment propensity score model, the dataset was split 80% and 20% into a training and test set, and the training set was further split 75% and 25% into a development and validation set for hyperparameter search. Optimal hyperparameters were selected using a grid search across 3 model types, logistic regression, random forests, and light gradient boosted machine models. The model with the highest area under the receiver operating characteristic curve after 3-fold cross-validation was chosen to generate the probabilities of being observed and propensity scores for the entire dataset. To avoid the undue influence of extreme propensity scores, we applied symmetric trimming and only included patients with propensity scores between 0.05 and 0.95. We also only included patients with follow-up time for the treatment outcome under consideration. After adjusting for the observation probability and propensity for treatment, the average treatment effect (ATE) was estimated as follows:



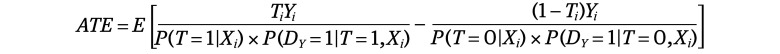



where *i* is the participant, *X* are the covariates, *T* = 1 if given a first-line treatment, *D_Y_* = 1 if the patient was followed for at least the outcome variable’s follow-up period, and *Y* is the outcome, which is treated as missing when *D_Y_* = 0. Confidence intervals for the propensity scores and ATE were generated using bootstrapping.^16^ Significance was defined as a 95% CI that did not cross the null value of 1. Feature importance for both models was determined using Shapley Additive Explanation values.^17^ eFigure 1 in Supplement 1 represents the analytic pipeline.

We assessed for residual confounding by estimating treatment effect on 3 negative control outcomes: fibrocystic disease of the breast, hernia, and fracture.^4,18^ These were selected on the basis of domain knowledge and a comprehensive literature search that found no evidence of a causal association with exposure to our antibiotics of interest. Treatment effect was calculated by estimating the prevalence of each negative control outcome at 1 month and 3 months after exposure.

Analyses were run separately for domain expert–derived and OMOP-learn–derived feature sets using the same analysis pipeline. All analyses were run in Python software version 3.85 (Python Software Foundation), and source code to reproduce all analyses is available at GitHub.^19^

## Results

### Baseline Cohort Description

**Figure 2.**
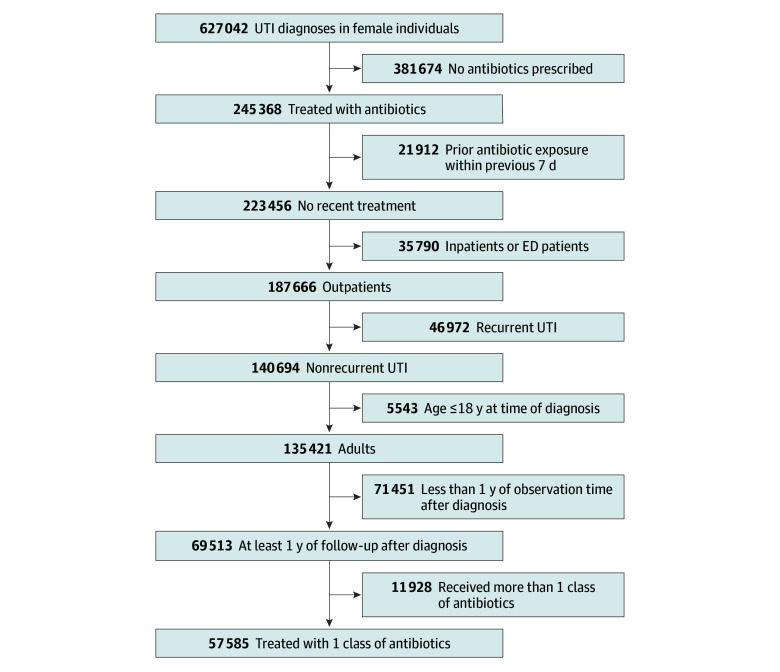
Study Flow Diagram Sample sizes indicate urinary tract infection (UTI) diagnoses. ED indicates emergency department.

The study flow diagram is shown in Figure 2, and baseline cohort characteristics are summarized in the Table. The final analysis cohort consisted of 57 585 episodes of UTI occurring in 49 037 female patients (mean [SD] age, 51.7 [20.1]) years. Of these, first-line antibiotics were prescribed in 35 018 episodes (61%), fluoroquinolones were prescribed in 21 140 episodes (37%), and β-lactams were prescribed in 1427 episodes (2%). Compared with those prescribed with first-line antibiotics, patients prescribed fluoroquinolones were older, more likely to have laboratory tests ordered, and had a higher comorbidity burden. Those prescribed β-lactams were similarly older and had a higher comorbidity burden. They were also more likely to be seen in the emergency department.

**Table.** Baseline Characteristics of Cohort

Characteristics	UTI diagnoses, No. (%)			
	Full cohort (N = 57 585)	First-line (n = 35 018)	Fluoroquinolones (n = 21 140)	β-lactams (n = 1427)
Age, mean (SD), y	51.7 (20.1)	48.5 (19.6)	56.5 (19.7)^a^	58.3 (21.6)^a^
Urinalysis ordered at presentation	13 713 (23.8)	7365 (21.0)	5937 (28.1)^a^	411 (28.8)^a^
Blood test ordered at presentation	2248 (3.9)	1040 (3.0)	1057 (5.0)^a^	151 (10.6)^a^
Menopause	3852 (6.7)	2320 (6.6)	1424 (6.7)	108 (7.6)
UTI in past year	5863 (10.2)	3418 (9.8)	2257 (10.7)^a^	188 (13.2)^a^
Underlying conditions				
Hypertension	21 026 (36.5)	10 742 (30.7)	9525 (45.1)^a^	759 (53.2)^a^
Diabetes	8298 (14.4)	4056 (11.6)	3910 (18.5)^a^	332 (23.3)^a^
Arthritis	11 220 (19.5)	6122 (17.5)	4698 (22.2)^a^	400 (28.0)^a^
Cancer	5684 (9.9)	2847 (8.1)	2639 (12.5)^a^	198 (13.9)^a^
Chronic kidney disease	2992 (5.2)	1364 (3.9)	1458 (6.9)^a^	170 (11.9)^a^
Autoimmune	2956 (5.1)	1639 (4.7)	1207 (5.7)^a^	110 (7.7)^a^
Thyroid disorder	292 (0.5)	148 (0.4)	136 (0.6)	8 (0.6)
Year of UTI episode				
2012-2014	7376 (12.8)	3435 (9.8)	3792 (17.9)^a^	149 (10.4)
2015-2017	26 770 (46.5)	14 757 (42.1)	11 420 (54.0)^a^	593 (41.6)
2018-2021	23 439 (40.7)	16 826 (48.1)	5928 (28.0)^a^	685 (48.0)
Physician specialty				
Family medicine	17 523 (30.4)	9710 (27.7)	7426 (35.1)^a^	387 (27.1)
Internal medicine	8025 (13.9)	3858 (11.0)	3930 (18.6)^a^	237 (16.6)^a^
Emergency care	4757 (8.3)	2968 (8.5)	1689 (8.0)^a^	100 (7.0)
Obstetrics and/or gynecology	2839 (4.9)	2124 (6.1)	684 (3.2)^a^	31 (2.2)^a^
Other nonurology specialty	6729 (11.7)	5119 (14.6)	1480 (7.0)^a^	130 (9.1)^a^
Urology	1498 (2.6)	907 (2.6)	522 (2.5)	69 (4.8)^a^
Others	2385 (4.1)	1433 (4.1)	818 (3.9)	134 (9.4)^a^

Abbreviation: UTI, urinary tract infection.

^a^ *P* < .05 vs first-line cohort.

### Primary Outcomes

For the domain expert–derived features, a light gradient boosting machine was the best model for estimating censorship, as well as the likelihood of treatment (details of performance are shown in the eAppendix and eFigures 2-8 in Supplement 1). The top 5 covariates estimating first-line vs fluoroquinolone therapy were year at UTI diagnosis, patient age, and physician specialty (ie, advanced specialist, internal medicine, or family medicine).

**Figure 3.**
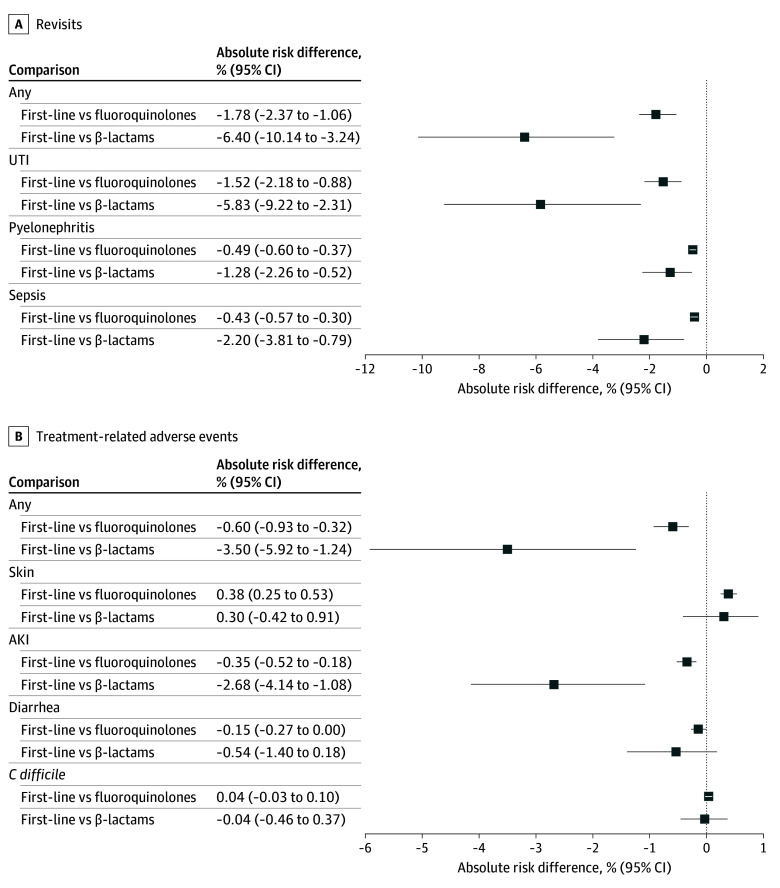
Primary and Secondary Outcomes Graphs show adjusted rate differences for revisits (A) and treatment-related adverse events (B) for patients receiving first-line vs fluoroquinolone antibiotics, and first-line vs β-lactam antibiotics, after adjusting for potential confounding factors and censoring. AKI indicates acute kidney injury; *C difficile*, *Clostridium difficile*; UTI, urinary tract infection.

Patients with UTI who were prescribed first-line antibiotics had a lower probability of an inpatient and outpatient revisit within 30 days compared with those who received a fluoroquinolone (adjusted risk difference, −1.78%; 95% CI, −2.37% to −1.06%). These results were primarily found in patients with uncomplicated UTI, but were also observed in those with pyelonephritis or sepsis to a lesser extent. Compared with β-lactam treatments, patients prescribed first-line antibiotics for UTI had a −6.40% (95% CI, −10.14% to −3.24%) lower probability of inpatient or outpatient revisit at 30 days (Figure 3A). For both comparisons, this finding was consistent regardless of whether the revisit was for UTI, pyelonephritis, or sepsis. We observed little to no difference in the subgroup analysis for patients with an inpatient revisit within 30 days (first-line vs fluoroquinolone adjusted risk difference, −0.14%; 95% CI, −0.29% to −0.02%; first-line vs β-lactam risk difference, 0.29%; 95% CI, −0.71% to 1.05%) (eFigure 4 in Supplement 1).

### Secondary Outcomes

In terms of adverse events, receipt of first-line antibiotics was associated with a slightly decreased risk of diarrhea (adjusted risk difference, −0.15%; 95% CI, −0.27% to 0.00%) and acute kidney injury (adjusted risk difference, −0.35%; 95% CI, −0.52% to −0.18%) vs fluoroquinolones. These patients had a higher risk of skin-related adverse events (adjusted risk difference, 0.38%; 95% CI, 0.25% to 0.53%). There was no difference in risk for *C difficile* infection between the treatment groups (Figure 3B).

Receipt of first-line antibiotics for UTI was associated with a lower risk of acute kidney injury (risk difference, −2.68%; 95% CI, −4.14% to −1.08%) vs receipt of a β-lactam (Figure 3B). There was no difference in the risk of a skin-related event, diarrhea, and *C difficile* infection.

### Negative Control Outcomes and Sensitivity Analyses

There were no differences in the 1-month and 3-month risk for the 3 negative control outcomes regardless of whether the patient received a first-line, fluoroquinolone, or β-lactam antibiotic, suggesting adequate adjustment for covariate-dependent censorship and for confounding by indication (eFigure 5 in Supplement 1). In addition, results obtained from the domain expert–derived feature set and the features derived from the OMOP-learn package were similar across all comparators and outcomes (eAppendix and eFigures 6-8 in Supplement 1). For the OMOP-learn model, compared with fluoroquinolones, first-line antibiotics were associated with a lower risk of medical revisits overall (−2.12%; 95% CI, −2.93% to −1.57%) and among those with inpatient revisits (−0.19%; 95% CI, −0.32% to −0.05%). They also were associated with a lower overall risk of any adverse events (−0.72%; 95% CI, −1.00% to −0.42%) but a higher rate of skin-related adverse events (adjusted risk difference, 0.33%; 95% CI, 0.17% to 0.44%). The comparison between first-line antibiotics and β-lactams using the OMOP-learn derived model were identical to the results found from the domain expert–derived model.

## Discussion

In this cohort study, using a large, contemporary clinical dataset, we demonstrate that IDSA guidelines for treatment of uncomplicated UTI remain robust in terms of both effectiveness and adverse events, despite major changes in the epidemiology of antibiotic resistance.^20,21^ Unless a patient has a history of drug resistance or intolerance or lives in a region where local rates of resistance are high, nitrofurantoin and trimethoprim-sulfamethoxazole remain the treatments of choice. We replicated our domain expert–derived results with an automated feature building package applied to a common data model, thereby supporting the hypothesis that complex causal inference analyses combined with careful cohort selection can be semiautomatable. This will help promote reproducibility of our findings in other health systems and opens inquiry into other important clinical questions.

We observed a small increase in rates of revisits for patients receiving fluoroquinolone therapy compared with those receiving first-line antibiotics. This result is surprising because fluoroquinolones are thought to be equivalent or superior to nitrofurantoin and trimethoprim-sulfamethoxazole in terms of clinical effectiveness.^22^ The differences were limited to outpatients with a diagnosis of lower UTI and were much less pronounced for inpatients, suggesting that the benefit of first-line treatments is restricted to classic presentations of uncomplicated UTI. Follow-up visits soon after treatment may be the result of drug intolerance, toxic effects, or selection of a drug to which an organism is resistant. The latter may be a possible explanation for why people treated with nitrofurantoin and trimethoprim-sulfamethoxazole had fewer revisits. Recent work^23^ has suggested that rates of resistance to nitrofurantoin remain low, despite its widespread use, and may be due to a high barrier to resistance. Although resistance to trimethoprim-sulfamethoxazole is more common, clinicians are less likely to use this drug on the basis of IDSA guidance^3^ that recommends avoiding it when rates of local resistance exceed 20%, which is a common scenario throughout the US. In contrast, resistance to fluoroquinolones is most often mediated by the accumulation of variants in a single gene, often in response to antibiotic exposure. Given the high rate of fluoroquinolone prescription in the community, this may increase the risk for prescribing an agent to which the organism is resistant. This is further complicated by the fact that uncomplicated UTI is often managed over telephone and without culture data. Finally, given that prescribers are prone to prescribe the same antibiotic,^24,25,26^ the impact of prior exposure may be more likely to lead to selection of resistance if that antibiotic is a fluoroquinolone and the patients are otherwise healthy outpatients with a low risk for colonization by drug-resistant organisms.

We applied 2 approaches to feature construction to correct for confounding. Domain expert–derived features are derived from expert knowledge on the biologic mechanisms of disease and real-world experience with managing uncomplicated UTI. These features have the advantage of theoretical backup from established pathophysiology and clinical data, but are limited by the possibility of missing potential confounders, especially when the disease has diverse mechanistic pathways or is not well understood. In contrast, OMOP-learn,^14^ which captures all information available in the data without prior knowledge of its relationship with the disease, lowers the probability of missing confounders but comes at the expense of including a large number of nonrelevant covariates. Our study provides an empirical demonstration that extracting features under the OMOP-learn framework can yield conclusions comparable to the domain expert–derived features, which supports application of causal inference methods using automatic feature generation in the medical context.

### Limitations and Strengths

This study has limitations that should be mentioned. We note differences in baseline characteristics between treatment groups; therefore, as with all observational studies, there is a possibility that our results may be biased owing to residual confounding. However, we believe the degree of confounding is small because we adjusted for both covariate-dependent censoring and treatment indication, which are the major forms of confounding we expect to impact our results. This is further supported by the results of the negative control outcome analysis, which shows an equal distribution of control outcomes between trial groups. The consistency in the strength and direction of our outcomes between domain expert–derived and OMOP-learn–derived features lends further strength to the validity of our findings. Finally, recent work has highlighted that carefully constructed retrospective cohorts with proper statistical adjustment can provide robust results that complement findings from prospective randomized clinical trials.^27^

Our study has several other limitations. We note that the prevalence of certain comorbidities is lower in our cohort than in the general population. This may partly reflect the limited scope of our data, which come from a single health insurer primarily based in Southeast Pennsylvania but may also reflect our inclusion criteria, which intentionally restricted our analyses to people with uncomplicated UTI. We also had limited data on patient race, ethnicity, and socioeconomic status, which precluded our ability to assess for fairness across diverse subpopulations. Future work should seek to reproduce our analysis using larger datasets with more diverse populations to ensure equity. The increase in prescription of first-line antibiotics over time, which likely reflects the effect of guideline dissemination and promotion of antibiotic stewardship,^28,29,30^ should not by itself bias outcomes, assuming care practices did not dramatically change over the study period.

The major strength of this study is the inclusion of a clinical dataset with a comprehensive collection of covariates translated into a common data model. The rich set of features permits construction of models that better capture causal mechanisms, and the use of a common data model enhances the study’s reproducibility for other patient populations. In addition, large observational datasets offer the opportunity to gain real-world insight that is both up to date and representative of the patients presenting with disease in practice today.

## Conclusions

In conclusion, our results provide reassurance that guideline-concordant therapy remains the optimal treatment decision for uncomplicated UTI. The application of an automated feature extraction package for datasets translated into a common data model, combined with a rigorous analytic pipeline, is a promising approach to assess the impact of guideline-directed therapy in real-world populations and over time.

## Notes

Supplement 1. **eAppendix**. Supplemental results **eTable 1.** List of *ICD-10-CM* diagnosis codes defining diagnosis of urinary tract infection **eTable 2.** List of procedures (*ICD-10-PCS*) and diagnoses (*ICD-10-CM*) codes for exclusion from the cohort **eTable 3.** List of conditions included in adverse event categories **eTable 4.** List of conditions and medications included as windowed history features in domain expert-derived features **eTable 5.** Prior antibiotic exposures for treatment groups **eFigure 1.** Analytic pipeline **eFigure 2.** Calibration plots for propensity for treatment and standardized mean differences before and after re-weighting for first-line antibiotics versus fluoroquinolones (A, C) and first-line antibiotics versus β-lactams (B, D) **eFigure 3. **Shapley value plots showing features ranked by absolute predictive importance for predicting receipt of (A) first-line versus fluoroquinolones and (B) first-line versus β-lactams, and impact of feature level for predicting receipt of (C) first-line versus fluoroquinolones and (D) first-line versus β-lactams **eFigure 4.** Adjusted rate difference for revisits resulting in inpatient admission for patients who received first-line versus fluoroquinolones, and first-line versus β-lactams, after adjusting for potential confounding factors and censoring **eFigure 5.** Adjusted rate difference for negative control outcomes for patients receiving first-line versus fluoroquinolones, and first-line versus β-lactams, after adjusting for potential confounding factors and censoring **eFigure 6.** Comparison of adjusted rate difference for treatment effectiveness using domain expert-derived features versus OMOP features, stratified by patients who received first-line versus fluoroquinolones, and first-line versus β-lactams **eFigure 7.** Comparison of adjusted rate difference for adverse events using domain expert-derived features versus OMOP features, stratified by first-line versus fluoroquinolones, and first-line versus β-lactams **eFigure 8.** Comparison of adjusted rate difference for negative control outcomes using domain expert-derived features versus OMOP features, stratified by first-line versus fluoroquinolones, and first-line versus β-lactams

Supplement 2.
Data Sharing Statement
