# Supplementary material for: Use of Machine Learning to Assess the Management of Uncomplicated Urinary Tract Infection
Source: JAMA Netw Open. 2025 Jan 31;8(1):e2456950. doi: 10.1001/jamanetworkopen.2024.56950 (PMC11786233; doi:10.1001/jamanetworkopen.2024.56950)
Supplement: Supplement 1. — eAppendix. Supplemental results eTable 1. List of ICD-10-CM diagnosis codes defining diagnosis of urinary tract infection eTable 2. List of procedures (ICD-10-PCS) and diagnoses (ICD-10-CM) codes for exclusion from the cohort eTable 3. List of conditions included in adverse event categories eTable 4. List of conditions and medications included as windowed history features in domain expert-derived features eTable 5. Prior antibiotic exposures for treatment groups eFigure 1. Analytic pipeline eFigure 2. Calibration plots for propensity for treatment and standardized mean differences before and after re-weighting for first-line antibiotics versus fluoroquinolones (A, C) and first-line antibiotics versus β-lactams (B, D) eFigure 3. Shapley value plots showing features ranked by absolute predictive importance for predicting receipt of (A) first-line versus fluoroquinolones and (B) first-line versus β-lactams, and impact of feature level for predicting receipt of (C) first-line versus fluoroquinolones and (D) first-line versus β-lactams eFigure 4. Adjusted rate difference for revisits resulting in inpatient admission for patients who received first-line versus fluoroquinolones, and first-line versus β-lactams, after adjusting for potential confounding factors and censoring eFigure 5. Adjusted rate difference for negative control outcomes for patients receiving first-line versus fluoroquinolones, and first-line versus β-lactams, after adjusting for potential confounding factors and censoring eFigure 6. Comparison of adjusted rate difference for treatment effectiveness using domain expert-derived features versus OMOP features, stratified by patients who received first-line versus fluoroquinolones, and first-line versus β-lactams eFigure 7. Comparison of adjusted rate difference for adverse events using domain expert-derived features versus OMOP features, stratified by first-line versus fluoroquinolones, and first-line versus β-lactams eFigure 8. Comparison of adjusted rate [file jamanetwopen-e2456950-s001.pdf]

Supplemental Online Content

Jones N, Shih MC, Healey E, et al. Use of machine learning to assess the management of uncomplicated urinary tract infection. *JAMA Netw Open*. 2025;8(1):e2456950. doi:10.1001/jamanetworkopen.2024.56950

eAppendix. Supplemental results

eTable 1. List of *ICD-10-CM* diagnosis codes defining diagnosis of urinary tract infection

eTable 2. List of procedures (*ICD-10-PCS*) and diagnoses (*ICD-10-CM*) codes for exclusion from the cohort

eTable 3. List of conditions included in adverse event categories

eTable 4. List of conditions and medications included as windowed history features in domain expert-derived features

eTable 5. Prior antibiotic exposures for treatment groups

eFigure 1. Analytic pipeline

eFigure 2. Calibration plots for propensity for treatment and standardized mean differences before and after re-weighting for first-line antibiotics versus fluoroquinolones (A, C) and first-line antibiotics versus β-lactams (B, D)

eFigure 3. Shapley value plots showing features ranked by absolute predictive importance for predicting receipt of (A) first-line versus fluoroquinolones and (B) first-line versus β-lactams, and impact of feature level for predicting receipt of (C) first-line versus fluoroquinolones and (D) first-line versus β-lactams

eFigure 4. Adjusted rate difference for revisits resulting in inpatient admission for patients who received first-line versus fluoroquinolones, and first-line versus β-lactams, after adjusting for potential confounding factors and censoring

eFigure 5. Adjusted rate difference for negative control outcomes for patients receiving first-line versus fluoroquinolones, and first-line versus β-lactams, after adjusting for potential confounding factors and censoring

eFigure 6. Comparison of adjusted rate difference for treatment effectiveness using domain expert-derived features versus OMOP features, stratified by patients who received first-line versus fluoroquinolones, and first-line versus β-lactams

eFigure 7. Comparison of adjusted rate difference for adverse events using domain expert-derived features versus OMOP features, stratified by first-line versus fluoroquinolones, and first-line versus β-lactams

eFigure 8. Comparison of adjusted rate difference for negative control outcomes using domain expert-derived features versus OMOP features, stratified by first-line versus fluoroquinolones, and first-line versus β-lactams

This supplemental material has been provided by the authors to give readers additional information about their work.

© 2025 Jones N et al. *JAMA Network Open*.

eAppendix. Supplemental results

Performance of propensity score and confounding models

First-line versus fluoroquinolones

For the domain expert-derived features, the best model predicting censorship for 15 and 90-day loss-to-follow up was a light gradient boosting machine, with AUROCs of 0.68, 0.66. The best censorship model for 30-day loss-to-follow up was a logistic regression with AUROC of 0.66. For predicting receipt of first-line or fluoroquinolones (to assess confounding by indication), the best model was also a light gradient boosting machine, with an AUROC of 0.73. Calibration curves are shown in eFigure 1A. The top 5 covariates contributing to the prediction include year at UTI diagnosis, patient age, and physician specialties (whether the physician was an advanced specialist, internal medicine or family medicine doctor).

First-line versus β-lactams

For the domain expert-derived features, the best model predicting censorship for 30 and 90-day loss-to-follow up was a light gradient boosting machine, with AUROC of 0.62 and 0.60. The best censorship model for 15-day loss-to-follow up was a random forest with AUROC of 0.64. For predicting receipt of first-line or β-lactams, the best model was a logistic regression, with AUROC 0.70 and calibration curve shown in eFigure 1B. Using SHAP values, the top 5 covariates contributing to the prediction include patient age, physician specialty in non-prespecified groups, hypertension history and alternative antibiotics history (6 months and 1-2 years).

© 2025 Jones N et al. *JAMA Network Open.*

Diagnosis description	ICD-10-CM code
Urinary tract infectious disease	N39.0
Acute cystitis	N30.00
Cystitis	N30.90
Hematuria co-occurrent and due to cystitis	N30.01
Hematuria co-occurrent and due to acute cystitis	N30.01

eTable 1. List of ICD-10-CM diagnosis codes defining diagnosis of urinary tract infection.

© 2025 Jones N et al. *JAMA Network Open.*

Excluded if occurred within 180 days before UTI diagnosis	
Pregnancy	Z33.1
Pyelonephritis	N12
Urinary catheterization	Z96.0
Procedures related to central venous catheter	Z95.828
Any surgery or mechanical ventilation	Z98.890, Z99.11
Hemodialysis	Z49.31
Parenteral nutrition	3E0336Z

Excluded if ever occurred before UTI diagnosis	
Neurogenic bladder	N31.9
Spina bifida	Q05.9
Malignancies of the urinary tract	C68.8
Gynecological Malignancies	C55

eTable 2. List of procedures (ICD-10-PCS) and diagnoses (ICD-10-CM) codes for exclusion from the cohort.

© 2025 Jones N et al. *JAMA Network Open.*

C. *difficile* infection	
Clostridium difficile colitis, *Clostridioides difficile* infection	A04.72

Skin	
Dermatitis due to drug AND/OR medicine taken internally	L27.0
Pruritic rash	L28.2
Urticaria	L50.9
Eruption	R21
Contact dermatitis due to drugs AND/OR medicine	L23.3

Gastrointestinal	
Diarrhea	R19.7

Acute kidney injury	
Acute renal failure syndrome	N17.9

eTable 3. List of conditions included in adverse event categories.

© 2025 Jones N et al. *JAMA Network Open.*

Diagnosis description	ICD-10-CM code
Addison’s disease	E27.1
Arthritis	M19.90
Catheter-related conditions	T80.219S
Celiac disease	K90.0
Chronic kidney disease	N18.9
Corticosteroid	T38.0X1A
Dermatomyositis	M33.90
Diabetes mellitus	E11.9
Graves disease	E05.00
Hashimoto thyroiditis	E06.3
Hemodialysis	Z99.2
HIV	Z21
Hypertension	I10
Lupus erythematosus	L93.0
Malignancies	C80.1
Menopause	Z78.0
Morbid obesity	E66.01
Multiple sclerosis	G35
Myasthenia gravis	G70.00
Pernicious anemia	D51.0
Reactive arthritis	M02.30
Rheumatoid arthritis	M06.9
Sjogren’s disease	M35.00
Transplantation	Z94.9
Urinary incontinence	R32

eTable 4. List of conditions and medications included as windowed history features in domain expert-derived features.

© 2025 Jones N et al. *JAMA Network Open.*

Treatment group	n	NIT	SXT	CIP	OFX	LVX	AMC	CPD	CFR	Any
All patients	57,585	4.5	4.6	5.5	0.5	2.2	5.2	0.0	0.2	19.3
First-line	35,018	5.2	4.8	4.1	0.5	1.6	5.1	0.0	0.2	18.3
Fluoroquinolones	21,140	3.3	4.2	7.6	0.4	3.2	5.0	0.0	0.1	20.4
β-lactams	1,427	5.5	4.8	6.6	0.6	3.4	11.0	0.2	0.5	26.6
NIT	20,064	6.3	3.7	4.3	0.5	1.4	5.2	0.0	0.2	18.2
SXT	14,954	3.7	6.3	4.0	0.4	1.8	5.0	0.0	0.2	18.4
CIP	18,593	3.2	4.1	7.7	0.4	2.5	5.0	0.0	0.1	19.8
OFX	17	11.8	0.0	11.8	0.0	0.0	11.8	0.0	0.0	29.4
LVX	2,530	4.1	4.9	7.3	0.5	8.3	4.9	0.1	0.2	24.5
AMC	1,235	5.8	5.0	6.5	0.7	3.5	12.2	0.0	0.1	27.5
CPD	124	1.6	4.0	7.3	0.0	3.2	3.2	2.4	0.0	16.9
CFR	68	8.8	2.9	7.4	0.0	2.9	2.9	0.0	8.8	27.9

eTable 5. Prior antibiotic exposures for treatment groups. Exposure represents the 6-month history for patients in the indicated treatment group.

Abbreviations

- NIT, nitrofurantoin
- SXT, trimethoprim-sulfamethoxazole
- CIP, ciprofloxacin
- OFX, ofloxacin
- LVX, levofloxacin
- AMC, amoxicillin-clavulanate
- CPD, cefpodoxime
- CFR, cefadroxil

© 2025 Jones N et al. *JAMA Network Open.*

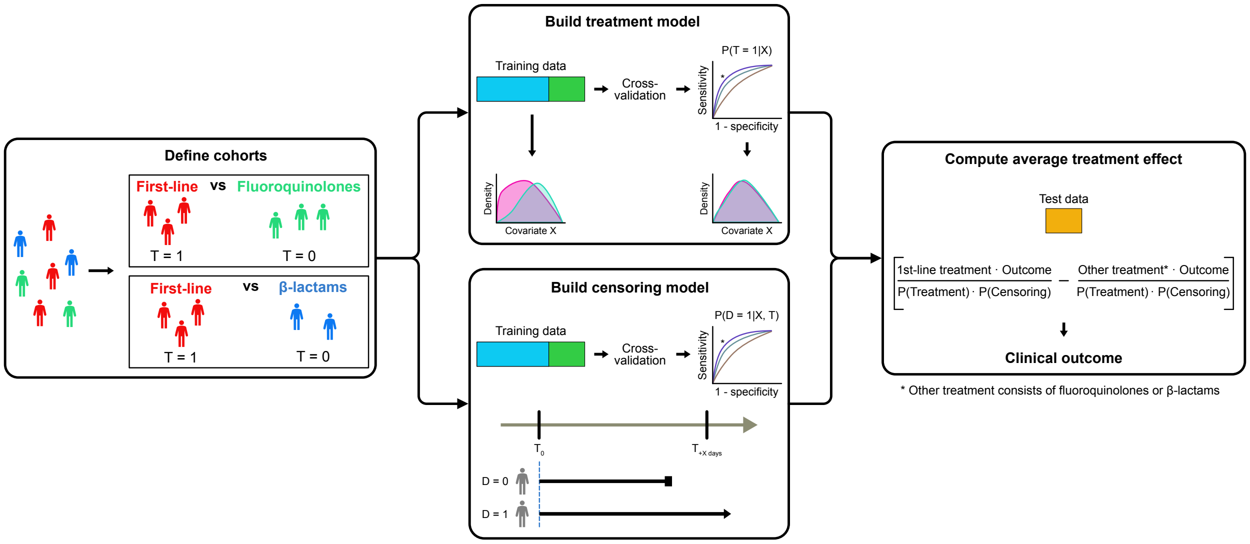

eFigure 1. Analytic pipeline. Separate cohorts were built for first-line versus fluoroquinolone and first-line versus β-lactam treatment. Eighty percent of the total data was set aside for training and this was further split 75/25 into development (blue) and validation (green) datasets. Two models were then run to estimate the probability of treatment and of being observed through the outcome period post-diagnosis. Three-fold cross-validation was used to select the model with the highest AUROC, indicated by the asterisk. Average treatment effect was estimated on test data (yellow) by the risk difference for a given outcome between those receiving first-line treatment or another treatment (fluoroquinolones or β-lactams) after normalizing for the probability of receiving a treatment and of being observed at the end of the outcome's follow-up period (e.g 30 days). Abbreviations, T, treatment, X, covariates, D, observed.

© 2025 Jones N et al. *JAMA Network Open.*

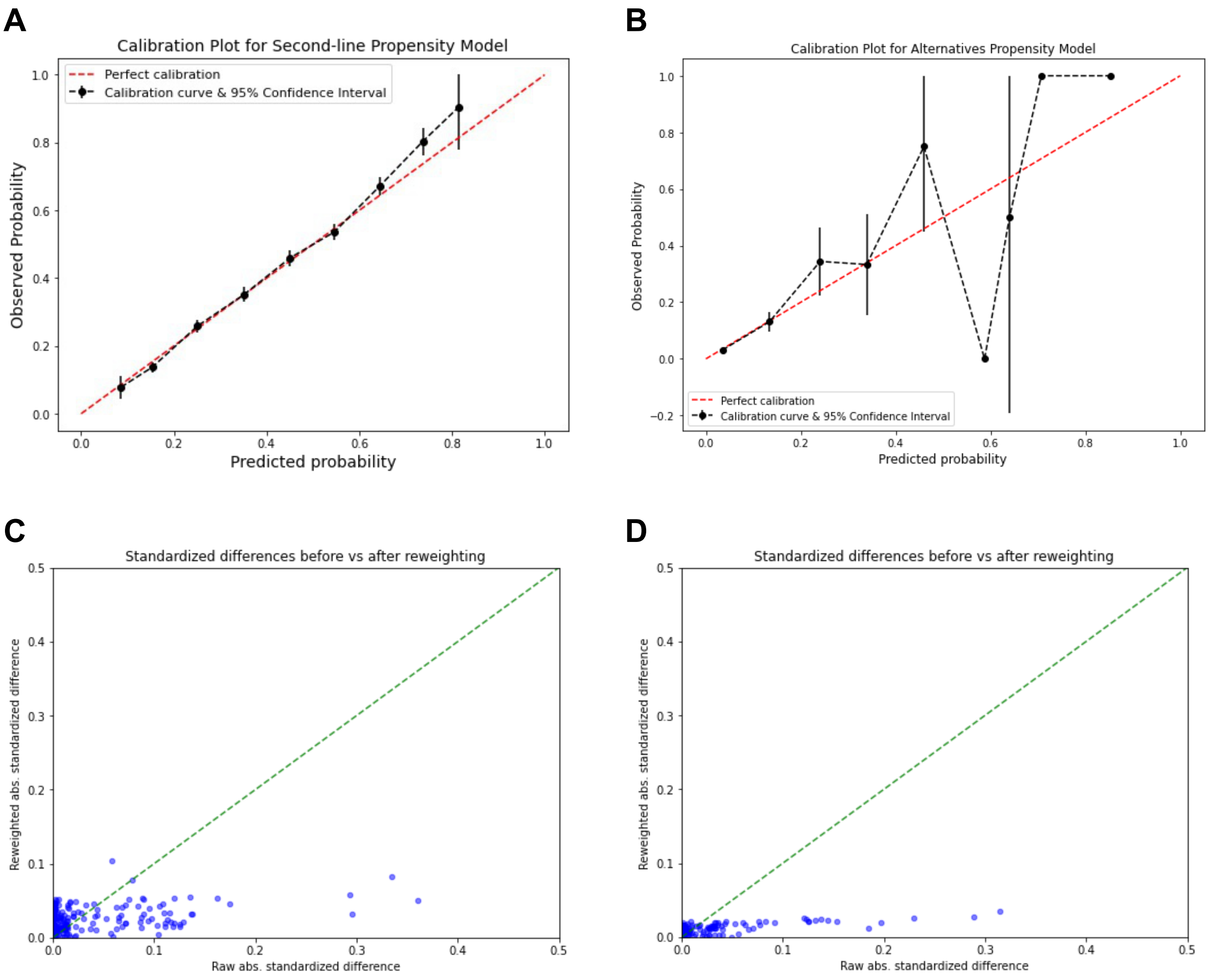

eFigure 2. Calibration plots for propensity for treatment and standardized mean differences before and after re-weighting for first-line antibiotics versus fluoroquinolones (A, C) and first-line antibiotics versus β-lactams (B, D). The calibration probability was calculated using validation data comprised of 20% of the dataset.

© 2025 Jones N et al. *JAMA Network Open.*

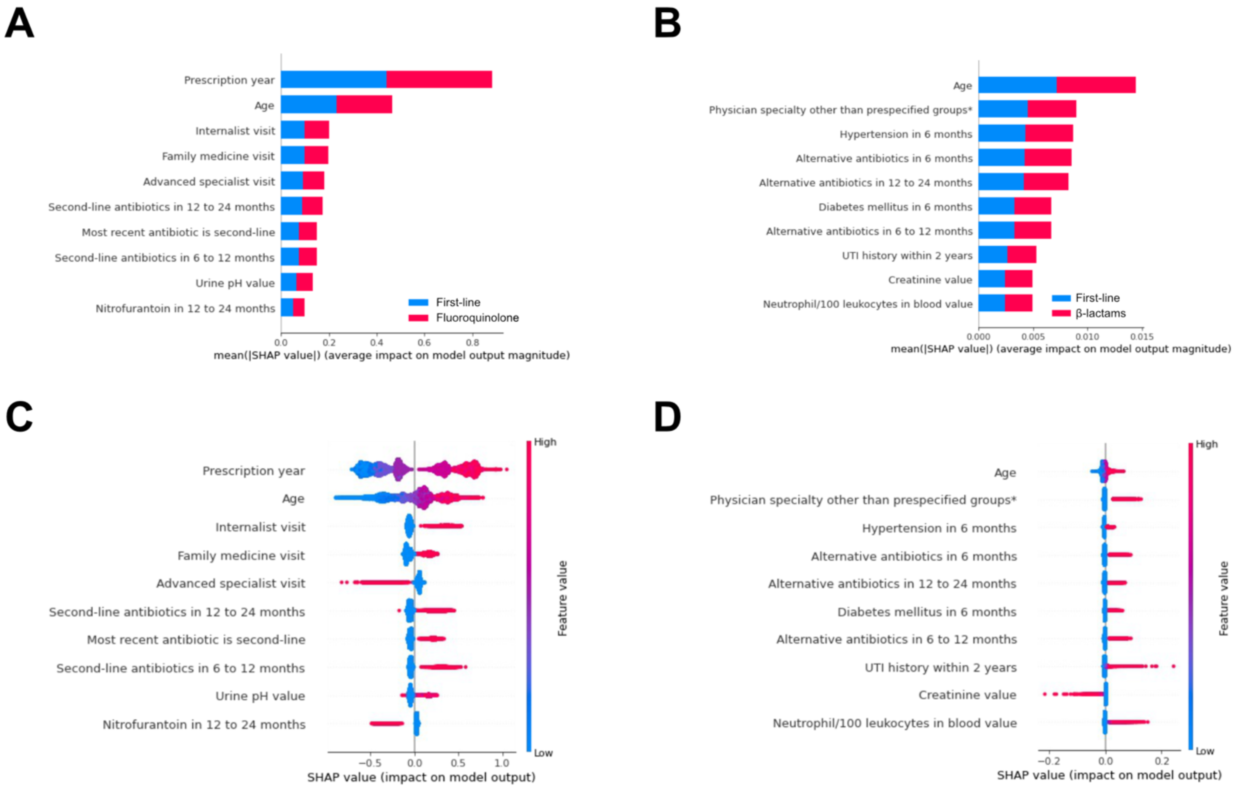

eFigure 3. Shapley value plots showing features ranked by absolute predictive importance for predicting receipt of (A) first-line versus fluoroquinolones and (B) first-line versus β-lactams, and impact of feature level for predicting receipt of (C) first-line versus fluoroquinolones and (D) first-line versus β-lactams.

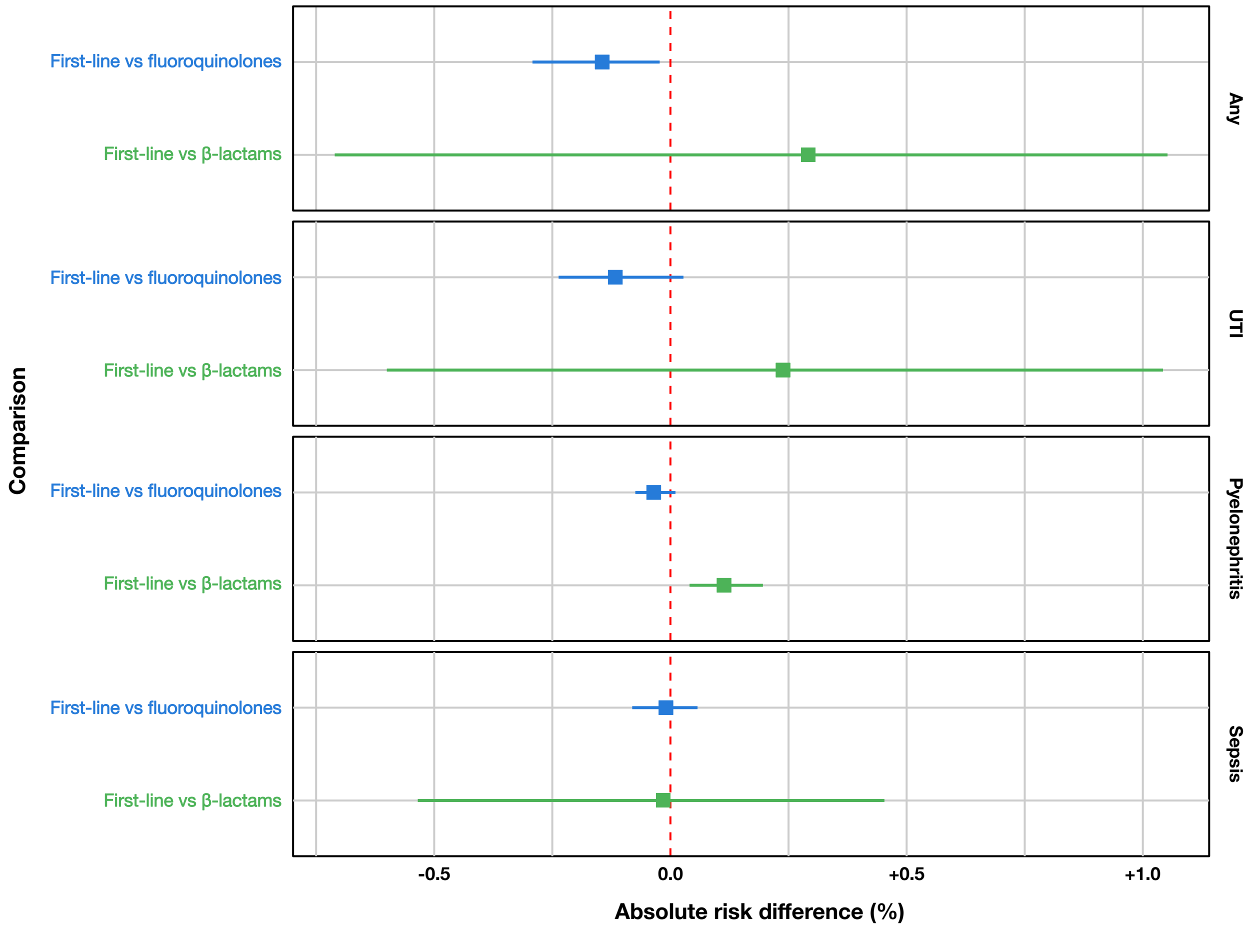

eFigure 4. Adjusted rate difference for revisits resulting in inpatient admission for patients who received first-line versus fluoroquinolones, and first-line versus β-lactams, after adjusting for potential confounding factors and censoring.

© 2025 Jones N et al. *JAMA Network Open.*

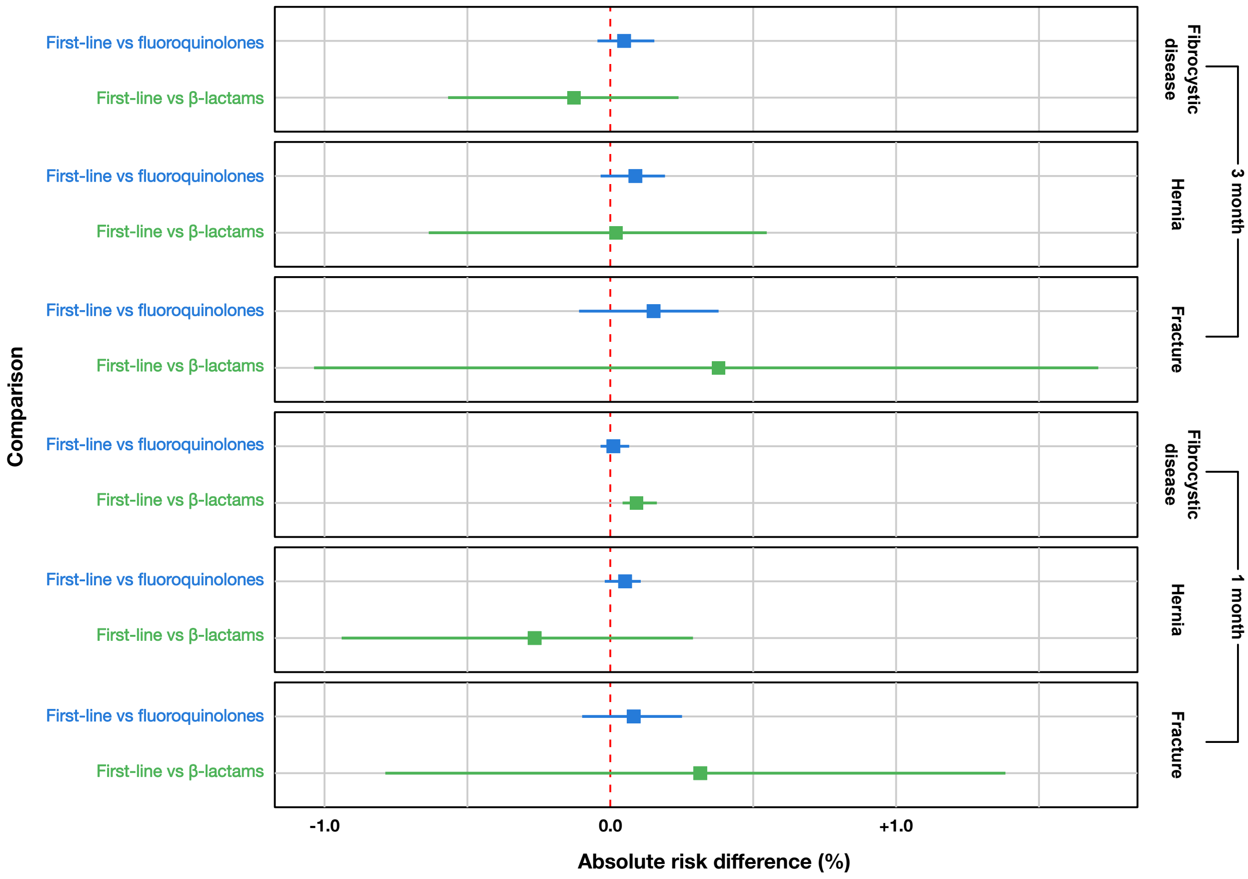

eFigure 5. Adjusted rate difference for negative control outcomes for patients receiving first-line versus fluoroquinolones, and first-line versus β-lactams, after adjusting for potential confounding factors and censoring.

© 2025 Jones N et al. *JAMA Network Open.*

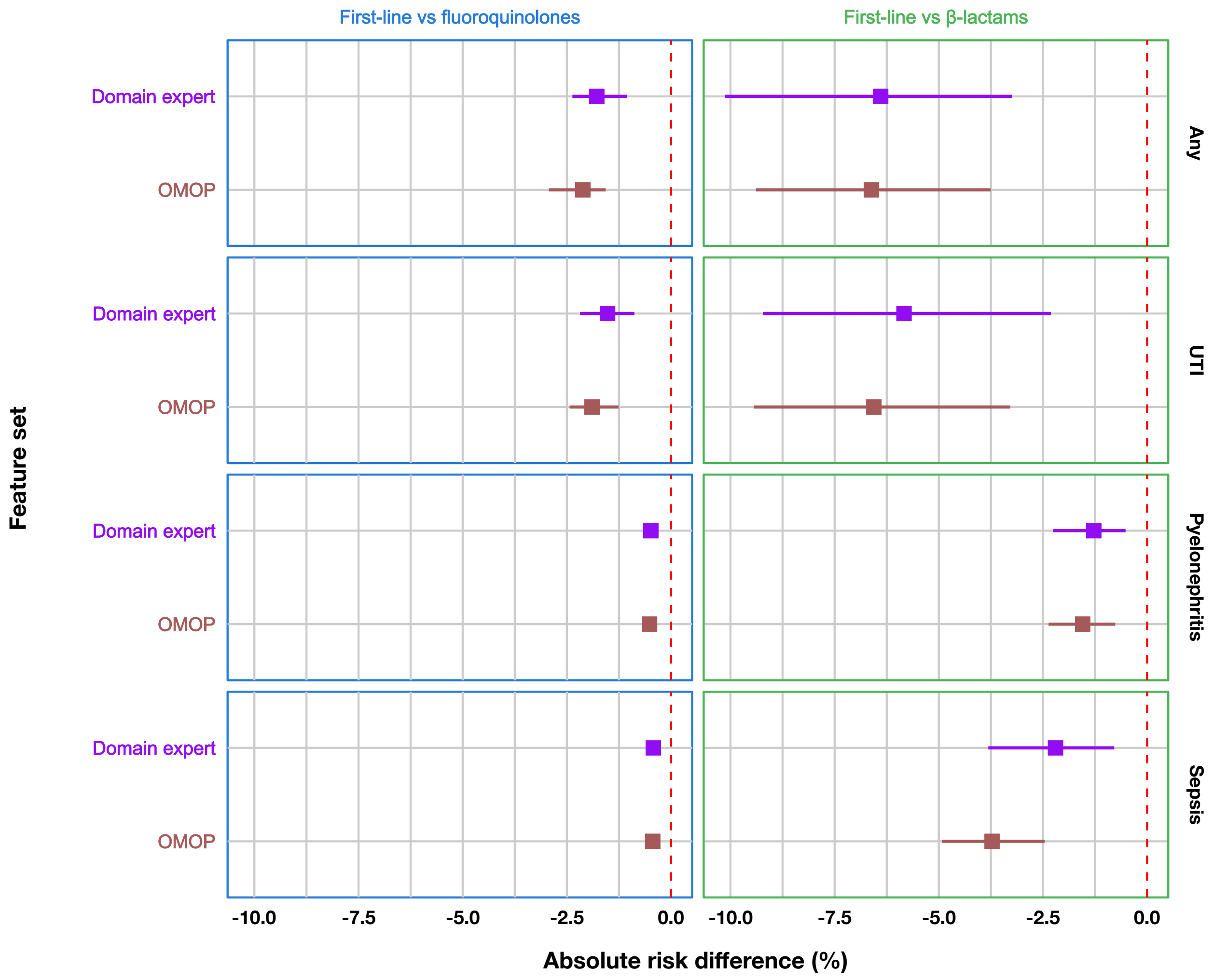

eFigure 6. Comparison of adjusted rate difference for treatment effectiveness using domain expert-derived features versus OMOP features, stratified by patients who received first-line versus fluoroquinolones, and first-line versus β-lactams. Treatment effectiveness was estimated by 30-day revisits overall, and for UTI, pyelonephritis and for sepsis.

© 2025 Jones N et al. *JAMA Network Open.*

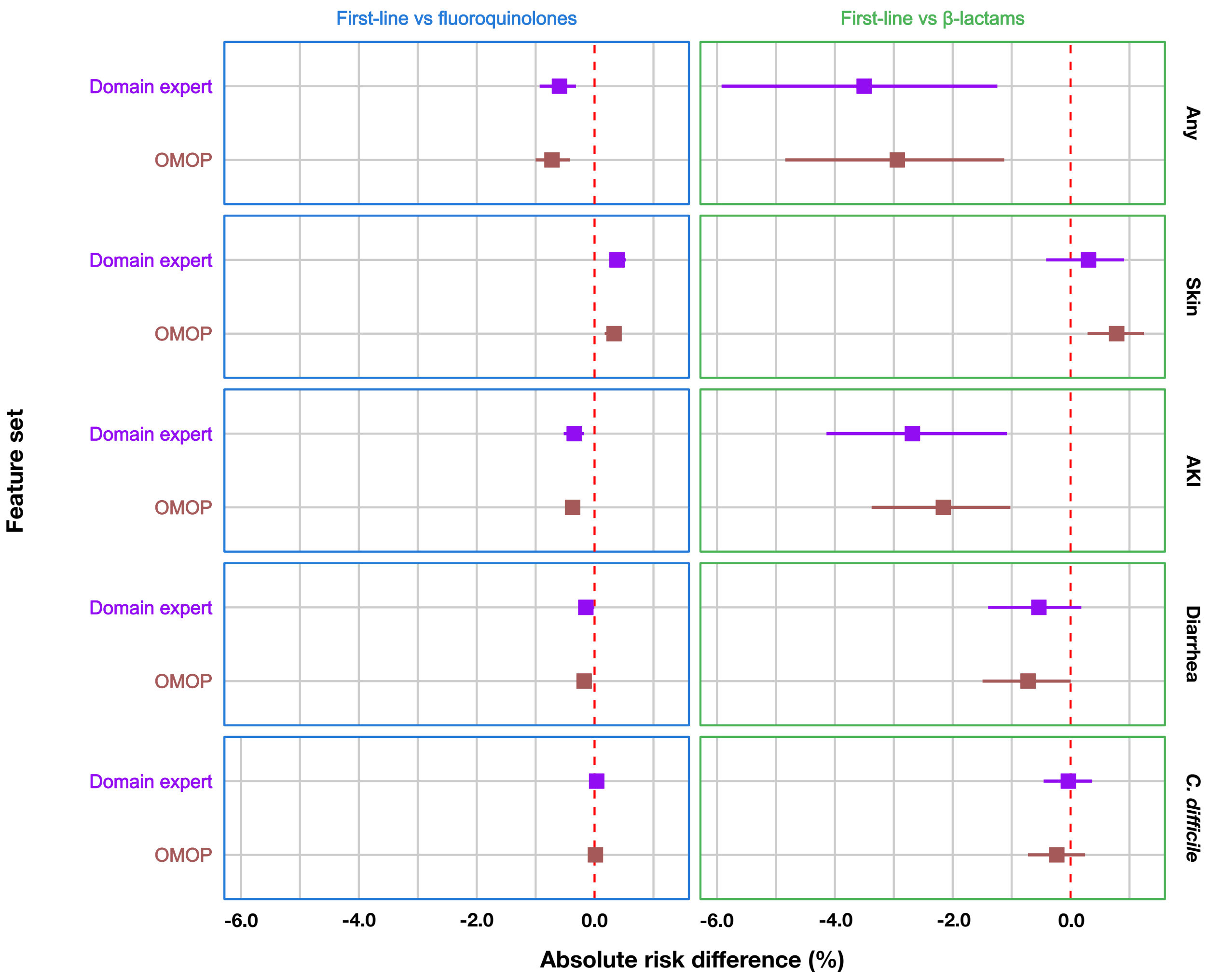

eFigure 7. Comparison of adjusted rate difference for adverse events using domain expert-derived features versus OMOP features, stratified by first-line versus fluoroquinolones, and first-line versus β-lactams.

© 2025 Jones N et al. *JAMA Network Open.*

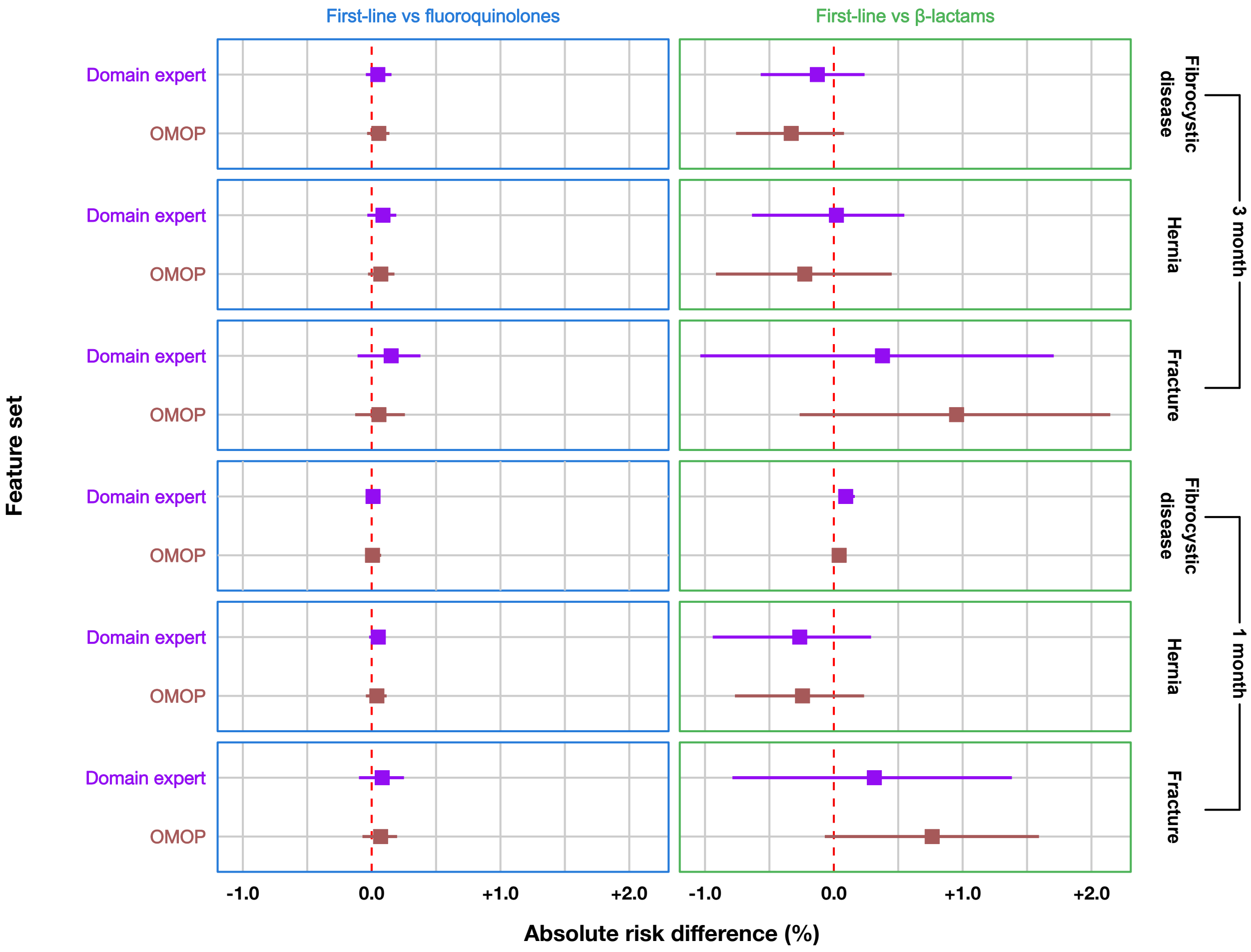

eFigure 8. Comparison of adjusted rate difference for negative control outcomes using domain expert-derived features versus OMOP features, stratified by first-line versus fluoroquinolones, and first-line versus β-lactams.

© 2025 Jones N et al. *JAMA Network Open.*
