# Supplementary material for: Use of Machine Learning to Assess the Management of Uncomplicated Urinary Tract Infection
Source: JAMA Netw Open. 2025 Jan 31;8(1):e2456950. doi: 10.1001/jamanetworkopen.2024.56950 (PMC11786233; doi:10.1001/jamanetworkopen.2024.56950)
Supplement: Supplement 2. — Data Sharing Statement [file jamanetwopen-e2456950-s002.pdf]

Data Sharing Statement

Data
Data available: No

Additional Information
Explanation for why data not available: Proprietary dataset containing protected health information belonging to Independence Blue Cross
